# Induction of Intestinal Th17 Cells by Flagellins From Segmented Filamentous Bacteria

**DOI:** 10.3389/fimmu.2019.02750

**Published:** 2019-11-22

**Authors:** Yanling Wang, Yeshi Yin, Xin Chen, Yongjia Zhao, Yichen Wu, Yifei Li, Xin Wang, Huahai Chen, Charlie Xiang

**Affiliations:** ^1^State Key Laboratory for Diagnosis and Treatment of Infectious Diseases, National Clinical Research Center for Infectious Diseases, Collaborative Innovation Center for Diagnosis and Treatment of Infectious Diseases, The First Affiliated Hospital, College of Medicine, Zhejiang University, Hangzhou, China; ^2^Key Laboratory of Comprehensive Utilization of Advantage Plants Resources in Hunan South, College of Chemistry and Bioengineering, Hunan University of Science and Engineering, Yongzhou, China; ^3^State Key Laboratory of Breeding Base for Zhejiang Sustainable Pest and Key Laboratory for Food Microbial Technology of Zhejiang Province, Zhejiang Academy of Agricultural Sciences, Hangzhou, China

**Keywords:** segmented filamentous bacteria, flagellin, Th17 cells, IL-17A, SI EC

## Abstract

T-helper-17 (Th17) cells are a subset of CD4+ T cells that can produce the cytokine interleukin (IL)-17 and play vital roles in protecting the host from bacterial and fungal infections, especially at the mucosal surface. These are abundant in the small intestinal lamina propria (SILP) and their differentiation are associated with the colonization of the intestinal flora. Segmented filamentous bacteria (SFB) drew the attention of researchers due to their unique ability to drive the accumulation of Th17 cells in the SI LP of mice. Recent work has highlighted that SFB used microbial adhesion-triggered endocytosis (MATE) to transfer SFB antigenic proteins into small intestinal epithelial cells (SI ECs) and modulate host immune homeostasis. However, which components of SFB are involved in this immune response process remains unclear. Here, we examined the roles of SFB flagellins in Th17 cells induction using various techniques, including ELISA, ELISPOT, and RNA-seq *in vitro* and *in vivo*. The results show that the immune function of SFB flagellins is similar to SFB, i.e., induces the appearance of CD4+ T helper cells that produce IL-17 and IL-22 (Th17 cells) in the SI LP. Furthermore, treatment of mice with SFB flagellins lead to a significant increase in the expression of genes associated with the IL-17 signaling pathway, such as IL-6, IL-1β, TNF-α, IL-17A, IL-17F, and IL-22. In addition, SFB flagellins have an intimate relationship with intestinal epithelial cells, influencing the expression of epithelial cell-specific genes such as Nos2, Duox2, Duoxa2, SAA3, Tat, and Lcn2. Thus, we propose that SFB flagellins play a significant role in the involvement of SFB in the induction of intestinal Th17 cells.

## Introduction

The gastrointestinal tract of vertebrates is colonized by a diverse array of microorganisms, that maintain a mutually beneficial relationship with the host (1). The important link of this relationship is based on the perception of specific bacterial species, that trigger responses required for maintaining homeostasis between microbiota and host (2). It is well-recognized that several individual bacterial species can affect the development and function of various immune cells and immune responses both in the gut and system (3). For instance, *Clostridium* can inhibit intestinal inflammation and IgE production through Foxp3+ regulatory T cells (4), and segmented filamentous bacteria (SFB) can induce Th17 cells differentiation in the small intestine (5, 6).

SFBs are spore-forming gram-positive bacteria with a segmented and filamentous morphology and primarily colonize the distal ileum of mice and rats (7). These bacteria tightly adhere to small intestinal epithelial cells (SI ECs), influencing the immune responses (5, 8). In particular, SFB induces the differentiation of Th17 cells that are characterized by the production of IL-17A, IL-17F, and IL-22. Th17 cell differentiation is controlled by the expression of RAR-related orphan receptor gt (RORgt) (9, 10). To date, the cytokines that can promote the differentiation of Th17 cells have been well-defined *in vitro* (9). IL-6, TGF-β, and IL-21 promote the differentiation of Th17 cells (11, 12). The coordinated activities of IL-1 and TNF can accelerate this process (13). In addition, cytokine IL-23 is not sufficient to generate Th17, but maintains the expansion and pathogenicity of Th17 cells (14).

At steady state, numerous Th17 cells are found in the small intestinal lamina propria (SILP), where they accumulate only in the presence of luminal commensal microbiota such as SFB (10). It has been suggested that the production of ATP and serum amyloid proteins induced by intestinal microorganisms could contribute to the generation of intestinal Th17 cells (5, 15). Furthermore, a recent report revealed that the microbiota could induce the production of IL-1β and that stimulation of IL-1β-IL-1R signaling is essential in promoting the differentiation of Th17 cell (16). The mechanisms by which SFB mediate the differentiation of intestinal Th17 cells have been elucidated. Unlike invasive pathogens, SFB tightly adhere to the IECs of the ileum and do not penetrate the IEC cytosol. Simultaneously, SFB use microbial adhesion-triggered endocytosis (MATE) to transfer T cell antigens into the SI ECs (17) and induce the secretion of SAAs, which act on CD11c+ cells to induce the production of IL-1β and other cytokines that shape the tissue microenvironment to potentiate the induction of Th17 cells (5, 18).

It is clear that SFB can promote the differentiation of Th17 cells, but which components of SFB are involved in this immune response process remains unclear. In addition, the difficulty to successfully isolate and culture SFB *in vitro* has hindered thorough investigations. Until recently, the complete genome sequence of mouse SFB and rat SFB has been published (19, 20). However, one major question remained: How does the microbiota induce Th17 cells? Most reported microbiota-immune effects are mediated by the recognition of microbes by PRRs such as Toll-like receptors (TLRs) (21). The microbial ligands recognized by TLRs are not unique to pathogens, however, and are produced by both pathogenic and commensal microorganisms. It is well-known that the bacterial flagella gene is an important functional gene that affects bacterial colonization and host immune regulation (22). When flagellin adheres to the base of the intestinal epithelium, it initiates an innate immune response and the flagellin-mediated proinflammatory response (23). In addition, studies have shown that bacterial flagellin are recognized by Toll-like receptor 5 (TLR5) (24). TLR5 detects flagellin via MyD88, resulting in the induction of proinflammatory cytokines, antimicrobial defenses, and antiapoptotic effects (25). The flagellin of *Salmonella enterica serovar* is encoded by the *fliC* and *fliB* genes, of which *fliC* is the primary gene (26). In addition, studies shown that *Salmonella* FliC could result in the production of cytokines and the activation of dendritic cell (DC) (27, 28). In addition, immunization of mice with *Salmonella* FliC causes a robust activation of immune cells (29).

The complete genome sequence of mouse SFB showed that SFB encoded more than 40 (3% of total) putative chemotaxis- and flagella-related proteins, and a complete set of genes for flagellar assembly was identified, although they have lost many enzymes for completing pathways essential for their growth and survival (20, 30). Furthermore, the contribution of SFB flagellins to the immune system due to its non-observability in electron microscope analysis remains unclear. Thus, our research group has been prompted to extensively study SFB flagellins. Furthermore, we previously reported that SFB widely express the flagellin protein and encode four types of flagellin, of which three, FliC2, FliC3, and FliC4, are capable of binding to the TLR5 receptors (31), as earlier described (19). Based on the findings of recent studies, we think it is necessary to further investigate the contribution of SFB flagellins. By studying the action of SFB flagellins on the intestinal tract, we found that SFB flagellins promote the production of cytokines, such as IL-17, IL-21, and IL-22, and activate IEC to secrete SAAs. In addition, the induction of Th17 cells by SFB is affected by these cytokines, which ultimately promote the production of Th17 cells (8). Therefore, we believe that SFB flagellins are likely to be important proteins for the adhesion of SFB to host cells and are recognized as antigens by host epithelial cells or SILP CD11c+ cell surface receptors and initiate subsequent signaling pathways, thus playing key roles in the induction of Th17 cells.

## Materials and Methods

### Animals

Five-week-old male C57BL/6J (B6) mice were obtained from the SLRC Laboratory Animal Center (Shanghai, China). These were randomly divided into control and experimental groups and housed under SPF conditions at the Laboratory Animal Center of Zhejiang University. All animal experiments were approved by the Ethics Committee of the First Affiliated Hospital, Zhejiang University.

### Heterologous Expression, Extraction, and Purification of the Flagellin Proteins

Heterologous expression, extraction, and purification of SFB-mFliC3, SFB-rFliC3, SFB-m5i-FliC3, and sal-FliC3 were performed as described elsewhere (31). Briefly, the QIAamp Fast DNA Stool Mini Kit (Qiagen, Germany) was used to extract rat and mouse bacterial genomic DNA. The SFB-specific PCR primers 779F and 1008R were used to detect SFB DNA (32). Furthermore, on the basis of the conserved region in the SFB flagellin gene sequences obtained in our previous study, we designed a pair of SFB fliC3-specific primers, fliC3 F and fliC3 R. SFB fliC3 genes were subcloned into the pET-28a vector. Then, IPTG (Sangon Biotech, China) was added to overexpress FliC3 proteins in chemically competent BL21(DE3) cells (Transgen Biotech, Beijing, China). BugBuster master mix (Merck Millipore, Germany) was used to extract total bacterial proteins, a His Bind purification kit (Merck Millipore, Germany) was employed to purify SFB FliC3 proteins, and Pierce™ High Capacity Endotoxin Removal Spin Columns (Thermo Scientific™, USA) were used to eliminate endotoxins in the protein samples. A bicinchoninic acid (BCA) protein assay kit (Beyotime Biotechnology, China) was used to determine the protein concentrations.

### LP Cell Isolation and *in vitro* Co-culture Experiments

Lamina propria lymphocytes (LPLs) were isolated as previously described, with minor modifications (33). Briefly, intestines were opened longitudinally and washed in ice-cold HBSS medium. Then, the intestinal tissues were cut into 1.5-cm segments and shaken twice for 25 min at 37°C in HBSS medium with 5% FBS (Gibco), 5 mM EDTA, and 1 mM DTT. After removal of the epithelial cells, the pieces were washed, minced, and shaken once for 45 min at 37°C in RPMI-1640 with 5% FBS, 1.5 mg/mL type VIII collagenase (Sigma-Aldrich), and 100 KU/l DNase I (Sigma). Then, the supernatants from the digestion were washed and resuspended in 40% Percoll (GE Healthcare, USA) and overlaid onto 80% Percoll in a 15-mL Falcon tube followed by centrifugation at 2,000 rpm for 20 min at room temperature. The LPLs were collected at the interphase of the Percoll gradient, washed, and resuspended in T cell medium. The cells were used immediately for subsequent experiments. LP CD4+ T cell subsets were first enriched by magnetic-activated cell sorting beads (MACS; Miltenyi Biotec) and then further purified with a FACSAria II (BD, USA). Approximately 5 × 10^4^ purified CD4 T cells were cocultured in 96-well U-bottom plates with 1 × 10^5^ MACS purified splenic CD11c+ cells as APCs in the presence or absence of flagellin proteins. After 24, 48, 72, and 96 h, the cell supernatant was collected, and IL-17A protein production was measured by ELISA (R&D Systems, USA).

### Cell Viability Assay

Cell viability was determined using a CCK-8 assay kit (Dojindo Laboratories, Kumamoto, Japan) according to the manufacturer's instructions. Briefly, 5 × 10^4^ CD4 T cells and 1 × 10^5^ splenic CD11c+ cells in 100 μL of culture media were plated to a 96-well U-bottom plates in the presence or absence of flagellin proteins. After 0, 24, 48, 72, and 96 h, CCK-8 (10 μL per well) reagent was added, and the reaction system was incubated for 1 h under the same incubator conditions. The relative viability of cells stimulated with different flagellin proteins was determined by measuring the absorbance of each well at a wavelength of 450 nm.

### IL-17A ELISPOT Assay

IL-17A ELISPOT was performed using a Mouse IL-17A FluoroSpot kit (Mabtech, Sweden). Briefly, an antibody solution was added to the plate and coated overnight at 4°C. Then, 5 × 10^4^ of purified CD4 T cells then were cocultured in pre-coated plates with 1 × 10^5^ MACS purified splenic CD11c+ cells as APCs in the presence or absence of flagellin proteins and incubated at 37°C in a humidified incubator with 5% CO_2_ for 72 h. In addition, spot analysis was performed with an automated fluorospot reader equipped with filters for the fluorophores used. The number of cells responding to flagellin protein stimulation was compared to the number of cells spontaneously secreting cytokines, which was determined by incubating the same number of cells in the absence of flagellin proteins.

### Flagellin Administration

Approximately 30 μg of purified SFB-mFliC3, SFB-rFliC3, SFB-m5i-FliC3, sal-FliC3, anti-CD3, or PBS were intraperitoneally injected into C57BL/6 mice (all mice were injected at a dose of 200 μL) (34). After 2 and 24 h, the ileal tissues and serum were collected for subsequent experiments or stored at −80°C.

### Cytokine Analysis by ELISA

The 1.5-cm small intestine tissues were cut from the distal ileum of each mice, opened longitudinally, washed in ice-cold PBS immediately, and weighed. Then, the tissues were lysed with T-PER™ Tissue Protein Extraction Reagent (Thermo Scientific™, USA) supplemented with protease inhibitors (Yeasen, China), followed by centrifugation at 1,500 *g* for 20 min at 4°C. The supernatants from the histiocyte lysates were collected and used immediately for subsequent experiments or stored at −80°C. IL-17A and IL-6 concentrations were measured using Mouse IL-17 Quantikine ELISA Kit and Mouse IL-6 Quantikine ELISA Kit (R&D Systems, USA). Cytokine concentrations in the tissues were expressed as amount per gram of tissue. Cytokine concentrations in the serum were expressed as amount per mL of serum.

### RNA-Seq and qPCR Analyses

Total RNA was isolated from small intestinal tissues and spleens using RNeasy Mini Kit (Qiagen, Germany), and total RNA was isolated from cocultures of CD4+ T cells and CD11c+ cells using iScript™ RT-qPCR Sample Preparation Reagent (Bio-Rad, USA). For real-time qPCR analysis, a PrimeScript™ RT reagent Kit (Takara, Japan) was used to synthesize cDNA, and qPCR was performed using SYBR® Premix Ex Taq™ (Tli RNaseH Plus) (Takara, Japan) on a 7500 Fast Real-Time PCR System (ABI). The primers used in the experiments are described in Supplementary Table 1. For RNA-seq analysis, RNA library preparation was performed using an Illumina TruseqTM RNA sample prep Kit. After assessing the library quality using TBS380 Picogreen, sequencing was conducted on an Illumina HiSeq2000. The sequence reads were mapped to the mouse reference genome using Tophat2 and Hisat2 software and normalized to Fragments Per Kilobase per Million mapped reads (FPKM) values. The raw RNA-seq datasets used in this study have been deposited in the NCBI Sequence Read Archive (http://www.ncbi.nlm.nih.gov/sra) as accession number PRJNA531884.

### Cell Culture and Flagellin Protein Stimulation of Epithelial Cell Lines

To detect stimulation of SFB flagellin proteins on a epithelial cell line, a method similar to a recent report (35, 36) was used. The mouse SI epithelial cell line MODE-K (BeNa Culture Collection, China) was maintained in RPMI-1640 with 10% FBS, 100 U/mL penicillin, and 100 μg/mL streptomycin solution. Purified SFB-mFliC3, SFB-rFliC3, SFB-m5i-FliC3, and sal-FliC3 (1 μg/mL) (37, 38) and phosphate-buffered saline (PBS) were added separately in MOED-K grown in 24-well plates and then incubated at 37°C for 24 h. The mRNA expression levels of SAAs and CCAAT-enhancer-binding protein (C/EBPD) were evaluated by qPCR.

### Western Blotting

Purified CD4 T cells, CD11c+ cells, and MODE-K cells were lysed in ice-cold RIPA lysis buffer (Beyotime Biotechnology, China) supplemented with protease inhibitor (Beyotime Biotechnology, China). BCA protein assay kit (Beyotime Biotechnology, China) was used to determine the protein concentrations. Equal amounts of total protein (25 μg) were separated by SDS-PAGE and the protein was transferred to polyvinylidene difluoride (PVDF) membranes (Millipore, USA). Following blocking with 0.5% bovine serum albumin, the membranes were incubated overnight with antibodies to GAPDH and TLR5 (Abcam, USA). Then the membranes were incubated with the secondary antibodies coupled to horseradish peroxidase. The Tanon-4500 gel imaging system was used to detect the protein bands (Tiangen, China).

### Application of Neutralizing Antibody

To neutralize TLR5, purified CD4 T cells, and CD11c+ cells were pre-incubated with an anti-TLR5 monoclonal antibody or the same concentration of monoclonal Rat IgG (InvivoGen, USA) for 1 h based on the manufacturer's directions. Cells were then treated with SFB flagellin proteins at 37°C for 3 days. The conditioned media were collected and analyzed for IL-17 using an ELISA kit as described above.

### Statistical Analysis

All of the statistical analyses were performed using GraphPad Prism and SPSS Software (version 22.0; SPSS Inc., USA) with two-tailed unpaired student's *t*-test or one-way ANOVA, followed by the appropriate *post-hoc* test. *P* < 0.05 were considered to be statistically significant.

## Results

### Induction of Th17 Cells by SFB Flagellins *in vitro*

**Figure 1:**
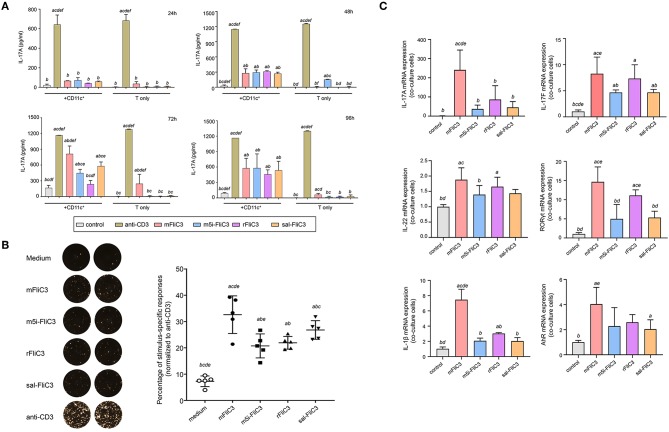
Cytokine IL-17A Production by Small Intestinal Lamina Propria Cells after stimulated *ex vivo* with SFB flagellins and other antigens. **(A)** Activation of SILP CD4+ T cells by SFB-mFliC3, SFB-rFliC3, SFB-m5i-FliC3, sal-FliC3, anti-CD3, or PBS. IL-17A ELISA assay was evaluated after 24, 48, 72, and 96 h. (a–f) Represent statistical significance relative to the control group; anti-CD3 group; mFliC3 group; m5i-FliC3 group; rFliC3 group; sal-FliC3 group, respectively. **(B)** IL-17A ELISPOT assay of SILP CD4+ T cells from WT mice treated with SFB flagellins and other antigens. (Left) Representative ELISPOT images. (Right) Compilation of results from multiple animals. Each symbol represents cells from a separate animal. **(C)** The mRNA expression of IL-17A, IL-17F, IL-22, RORγt, AhR, and IL-1β relative to Gapdh in a co-culture system stimulated with SFB flagellins. Data are expressed as the mean ± SEM of three independent experiments. Error bars indicate median values. (a–e) Represent statistical significance relative to the control group; mFliC3 group; m5i-FliC3 group; rFliC3 group; sal-FliC3 group, respectively.

Studies have shown that bacterial flagellins play a key role in the process of bacterial colonization and host immune regulation (22). To investigate whether SFB flagellins stimulate immune cells and participate in host immune regulatory response, CD4+ T cells, which were purified from small intestinal lamina propria, were cocultured with splenic CD11c+ cells as APCs and stimulated *ex vivo* either with anti-CD3 or with purified SFB-mFliC3, SFB-rFliC3, SFB-m5i-FliC3, and sal-FliC3. Cytokine expression was analyzed after stimulated for 24, 48, 72, and 96 h. As shown in Figure 1A, although less efficient than anti-CD3, all of these SFB flagellins and sal-FliC3 induced IL-17 expression in a significant number of CD4+ cells. In the case of SFB-mFliC3, the concentration of IL-17A gradually increased with time and the activation effect of CD4+ T cells was the best at about 72 h. Furthermore, the addition of SFB flagellins or sal-FliC3 to cultures with only T cells did not promote the production of Th17 cell cytokines. We conclude that the presentation of SFB flagellin-derived antigens by DCs such as CD11c+ cells is necessary and promotes the generation of Th17 cells. However, liquid chromatography-tandem mass spectrometry (LC-MS/MS) showed that a small amount of contaminating *E. coli* proteins from chemically competent BL21(DE3) cells were in the purified FliC3 (data not shown). To prove that FliC3, rather than contaminating *E. coli* proteins, induce Th17 cell cytokines, we used the same method to purify the empty BL21(DE3) cells to obtain the contaminating *E. coli* proteins. The co-culture system of the SILP CD4+ T cells and CD11c+ cells were then stimulated with purified contaminating *E. coli* proteins. We found that contaminating *E. coli* proteins do not promote the differentiation of Th17 cells (Supplementary Figure 1A).

Next, to estimate the cytotoxicity of SFB flagellins and sal-FliC3 over time, a CCK-8 assay was performed to quantify the cell viability of SFB flagellins and sal-FliC3-treated T cells. Supplementary Figure 1B shows the cell viability of different SFB flagellins and sal-FliC3-treated T cells after incubation for 0, 24, 48, 72, and 96 h. No significant differences in the relative viability of different SFB flagellins and sal-FliC3-treated T cells to control cells were observed. These results suggested that SFB flagellins had no obvious cytotoxic effects on these cells using our experimental conditions. To more accurately observe the expression of IL-17A at the single-cell level, an IL-17A ELISPOT assay was used to quantify the percentage of Th17 responding to these flagellin antigens (Figure 1B). Our results further indicated that SFB flagellins could stimulate immune cells, promoting the differentiation of CD4+ T cells. Anti-CD3 was assumed to activate 100% CD4 T cells, and we found that SFB-mFliC3 activated 30–40% of the CD4 T cells, and SFB-rFliC3, SFB-m5i-FliC3, and sal-FliC3 activated 20–30% of the CD4 T cells. We also assessed the influence of SFB flagellins on Th17 cell differentiation *in vitro* by quantitative RT-PCR. The addition of SFB flagellins to cocultures of CD4+ T cells and CD11c+ cells promoted the differentiation of Th17 cells, including IL-17A, IL-17F, IL-22, and RORgt. In addition to the increase in Th17 cell effector cytokines, the mRNA levels of the IL-1β and AhR were also highly upregulated in SFB flagellin-treated cells (Figure 1C). However, no remarkable changes were observed in relation to the mRNA levels of IL-21, IL-23, and TGF-β, which were defined to promote the differentiation of Th17 cells (Supplementary Figure 1C). In addition, the expression of Th1 cell-related cytokines in SFB flagellin-treated cells such as IFN-γ and T-bet, were not significantly enhanced (Supplementary Figures 1D,E).

### Induction of Th17 Cells by SFB Flagellins *in vivo*

**Figure 2:**
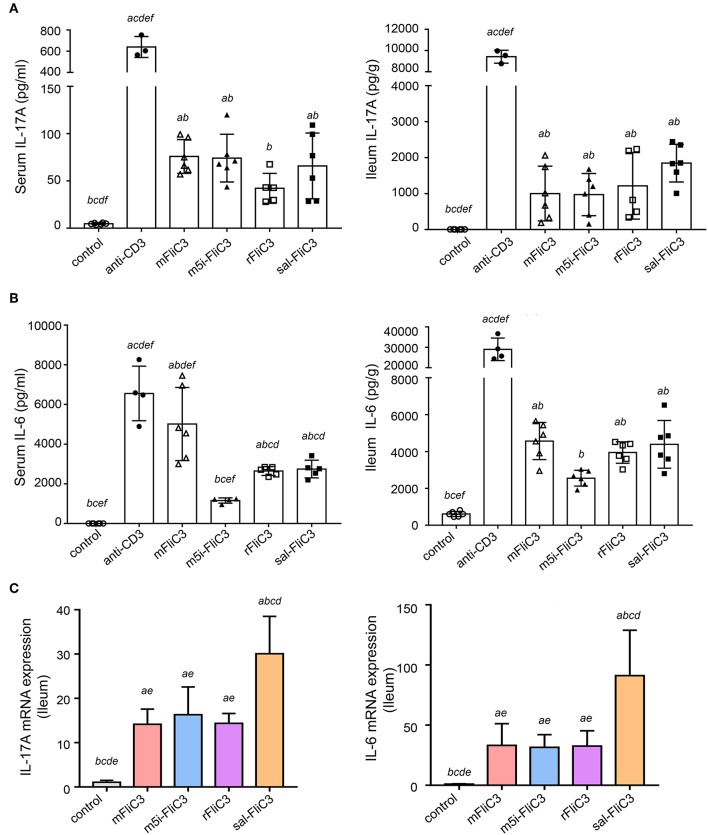
SFB flagellins promote a swift, transient, Th17-related response *in vivo*. Mice (*n* = 6) were i.p. injected with SFB flagellins or other antigens. The distal ileum and serum cytokine were assayed 2 h later. **(A)** Cytokine IL-17A concentrations detected in serum and supernatants of the distal ileum isolated from either mouse, which were i.p. with SFB flagellins or other antigens for 2 h (*n* = 6). **(B)** Serum and intestinal IL-6 concentrations in conventional and experimental mice that received i.p. immunizations for 2 h (*n* = 6). See statistical significance in Figure 1A. **(C)** The mRNA expression of IL-17A, IL-6 relative to Gapdh in intestinal of the indicated mice. Data are expressed as the mean ± SEM of three independent experiments. Error bars indicate median values. See statistical significance in Figure 1C.

To further prove that SFB flagellins can induce Th17 cells differentiation *in vivo*, 5-week-old male mice were intraperitoneally injected with SFB-mFliC3, SFB-rFliC3, SFB-m5i-FliC3, sal-FliC3, anti-CD3, or PBS. IL-17A and IL-6 concentration was analyzed after stimulation for 2 and 24 h. The cytokine concentration of mice immunized with SFB flagellins, compared with mice intraperitoneally injected with PBS, had significant changes, under the same feeding conditions. We found that when mice were intraperitoneally injected with SFB flagellins for 2 h, the concentration of IL-17A significantly increased in both the small intestine and serum (Figure 2A). Consistent with this result, the IL-6 concentration, which was well-acknowledged as a factor of contributing to the Th17 differentiation, was also significantly increased in the small intestine and serum (Figure 2B). However, although IL-6 and IL-17A concentration was still at a high level in mice injected with anti-CD3 for 24 h, either in the small intestine or in the serum, they all returned to normal level in the mice with flagellin administration (Supplementary Figures 2A,B). Meanwhile, the transcription of IL-17A and IL-6 in the small intestine was then monitored. As we saw, flagellin administration for 2 h triggered about a 15- to 20-fold increase of IL-17A mRNA levels and the mRNA level of IL-6, which promotes Th17 cell differentiation was significantly upregulated by 40- to 50-fold (Figure 2C). However, cytokine levels in the small intestine of the mice that received flagellins for 24 h returned to normal levels (Supplementary Figure 2C). In conclusion, we further evidenced the new point that SFB flagellins can promote the differentiation of Th17 cells by animal *in vivo* experiments and *in vitro* cell co-culture experiments.

### Influence of SFB Flagellins on the Intestinal Gene Expression Profiles of Mice

**Figure 3:**
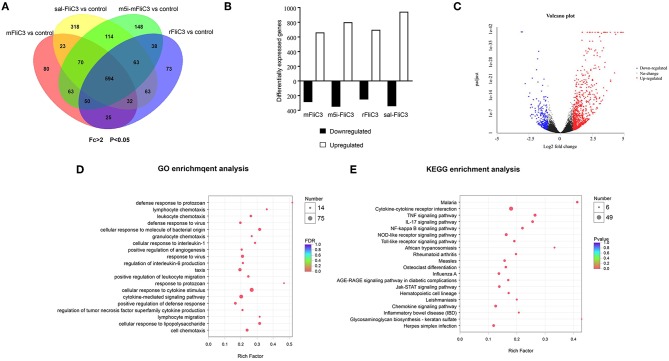
Transcriptional programs induced by SFB flagellins. **(A)** Venn diagrams showing the overlap between genes as influenced by either SFB-mFliC3, SFB-rFliC3, SFB-m5i-FliC3, or sal-FliC3 stimulation of mice (*n* = 3/group, *p* < 0.05). **(B)** RNA-seq analysis of differentially expressed genes in SFB-mFliC3, SFB-rFliC3, SFB-m5i-FliC3, and sal-FliC3 stimulation of mice relative to conventional mice (*n* = 3/group). Bar graphs represent number of genes higher expressed (open bar; fold-change > 2, *p* < 0.05) and lower expressed (black bar; fold change <-2, *p* < 0.05) in each treatment relative to the control group after 2 h of stimulation. **(C)** Volcano plot shows gene expression differences between SFB-mFliC3 and controls. Each point represents a gene from the uniquely common set of 51,912 genes between platforms. Volcano plot distributions of fold change (log2 [fold change]) (X-axis) and student's *t*-test *p*-values (–log10 [*p*-value]) (Y-axis) **(D,E)**, GO and KEGG enrichment analyses between small intestinal in SFB-mFliC3 stimulated mice vs. wild-type (WT) mice (2 h after inoculation).

**Figure 4:**
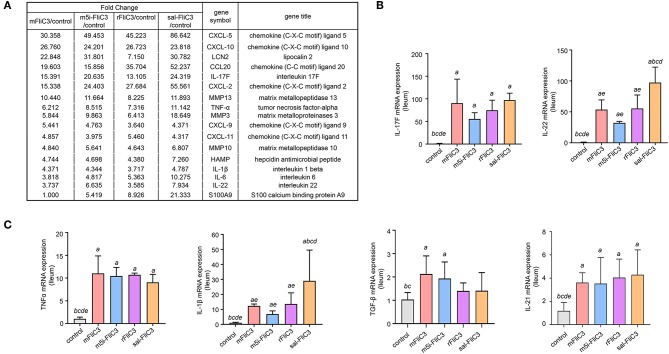
Enteric IL-17 signaling activation mediated by SFB flagellins. **(A)** The comparisons of gene expression specific for Th17-related response arranged by fold change in control and experimental groups after RNA-seq. **(B,C)** qPCR for IL-17F, IL-21, IL-22, TGF-β, TNF-α, and IL-1β relative to Gapdh in the intestines of the indicated mice that were i.p. injected with SFB flagellins or other antigens for 2 h (*n* = 3). Error bars indicate median values. See statistical significance in Figure 1C.

To determine the influence of SFB flagellins host gene expression, we compared the gene expression profiles in the ileum of C57BL/6 mice after administration of SFB-mFliC3, SFB-rFliC3, SFB-m5i-FliC3, and sal-FliC3 with PBS. We found that administration of mice with SFB-mFliC3, SFB-m5i-FliC3, SFB-rFliC3, or sal-FliC3 induced a significant change in the expression of 937, 1,140, 938, and 1,277 genes, respectively. In addition, these four gene sets showed fraction overlap, which contained 594 genes (Figures 3A,B). Among them, we emphatically distinguished genetic profiles differences between SFB-mFliC3 treatment group and PBS control group. As shown in Figure 3C, numerous (>70%) of these differentially expressed genes were upregulated and a handful of (>30%) were downregulated in SFB-mFliC3 treatment group compared to the PBS control group. GO and KEGG enrichment analyses of upregulated genes showed that SFB-mFliC3 significantly induced the immune system pathways. Furthermore, the chemokine signaling pathway, Toll-like receptor signaling pathway, and NF-kappa B signaling pathway, which indirectly promote the immune regulatory response and induce the differentiation of intestinal Th17 cells, had significantly changed (Figures 3D,E). We also found that SFB-mFliC3 administration of mice induced a significant change in expression of 118 immune system-related genes (Supplementary Figure 3). To assess significant changes associated with Th17 cells induced by SFB-mFliC3, we focused on the expression of genes specific for IL-17-mediated signaling (Figure 4A and Supplementary Figure 4). Gene profiling indicated that the expression of genes specific to IL-17-mediated signaling, including Th17-promoting cytokines, chemokines, and antimicrobial molecules, such as HAMP were significantly enhanced. Furthermore, the expression of the chemokine CCL20 was also significantly upregulated. However, no remarkable changes in the expression of IL-23 and AhR were observed (Supplementary Figure 5A). To verify the reliability of the intestinal gene expression profiles of the mice, the expression of Th17-promoting cytokines and Th17 effector cytokines mRNAs, including TNF-α, TGF-β, IL-1β, IL-21, IL-17F, and IL-22, were confirmed by quantitative RT-PCR (Figures 4B,C). Concordant with the gene expression profiles, the expression of TNF-α, IL-1β, IL-17F, and IL-22 were significantly upregulated, and the expression of IL-21 and TGF-β slightly changed. However, the mRNA levels of Th17-promoting cytokine and Th17 effector cytokine mRNAs returned to baseline levels after 24 h (Supplementary Figure 5B). Thus, we inferred that the immune regulatory response induced by SFB flagellins was temporary. We also verified the expression of Th17 cell-related cytokines, including IL-17A, IL-17F, IL-22, IL-6, IL-21, and TNF-α, in the spleen (Supplementary Figure 5C). The mRNA levels of Th17 cell-related cytokines were not significantly enhanced in the spleen. In summary, SFB flagellins promote the production of the innate cytokines IL-17A, IL-17F, and IL-22 in the small intestine, a pattern that resembles a Th17 related innate response.

### SFB-Flagellins Activate SI EC

**Figure 5:**
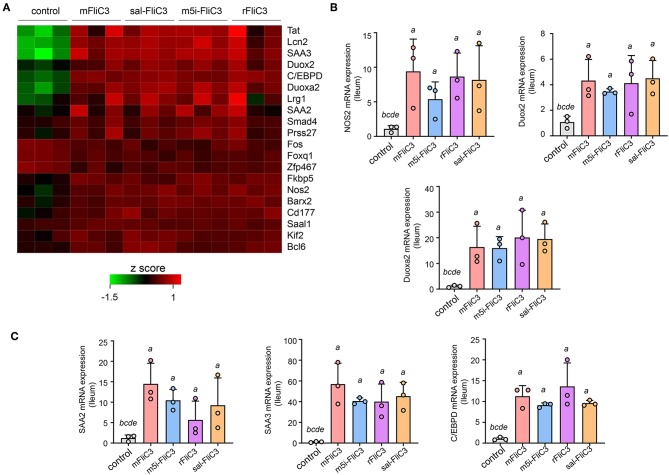
SFB flagellin-mediated EC activation. **(A)** Heat map showing the relative abundance for gene transcripts significantly expressed in flagellin-treated mice vs. conventional mice. **(B,C)** qPCR for the selected genes relative to Gapdh. Error bars indicate median values. See statistical significance in Figure 1C.

**Figure 6:**
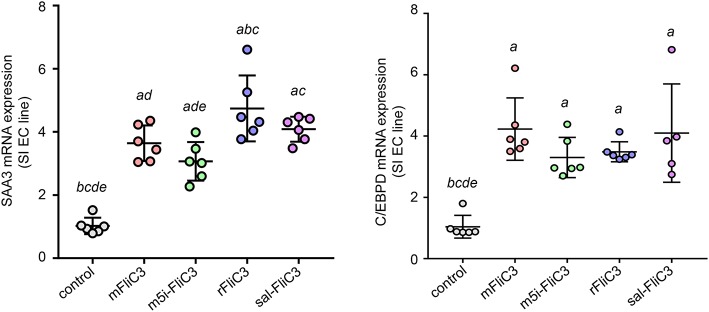
A unique gene expression pattern of IEC stimulated by SFB flagellins. MODE-K cells were stimulated by SFB flagellins for 24 h. The mRNA levels of SAAs and Cebpd were evaluated by qPCR. Error bars indicate median values. See statistical significance in Figure 1C.

Studies have confirmed that SFB induced Th17 cell differentiation in the intestine, mainly due to the interaction between SFB and the IECs, thus generating an environment conducive to Th17 cell differentiation (35). To examine whether SFB flagellins were involved in these unique signaling pathways, we examined the effects of SFB flagellins on SI EC gene expression profiles (Figure 5A). The expression of ROS-generating enzyme dual oxidase 2 (Duox2), its maturation factor Duoxa2 and nitric oxide synthase 2 (Nos2), were highly upregulated in SI ECs of mice intraperitoneally injected with SFB flagellins compared to conventional mice. In addition, the mRNA levels of these genes were verified by qPCR analysis (Figure 5B). Studies have shown that SFB colonization induces the secretion of SAAs, which act on CD11c+ cells to induce the production of IL-1β and other cytokines that shape the tissue microenvironment to potentiate the induction of Th17 cells (5, 35). Interestingly, two subtypes of serum amyloid A (SAA2 and SAA3) were significantly upregulated in the intestines of mice intraperitoneally injected with SFB flagellins (Figure 5C). However, there was no significant change in the expression of SAA1. A recent study suggested that C/EBPD could interact with both regulatory regions of SAAs (35). All results indicated that the mRNA levels of C/EBPD were highly upregulated (Figure 5C). We next examined the expression of SAAs and C/EBPD in a mouse SI EC line (MODE-K) stimulated with SFB flagellins *in vitro*. In line with RNA-seq, the co-induction of SAA3 and C/EBPD expression was observed (Figure 6). However, no differences in the expression of SAA1 and SAA2 were observed (Supplementary Figure 6).

## Discussion

SFBs play a significant role in promoting the differentiation of intestinal Th17 cells (6, 39). Here, a variety of experimental methods were used to prove that SFB flagellins can significantly induce mucosal expression of Th17-related cytokines, such as IL-17 and IL-22. Studies have indicated that *Salmonella* FliC could lead to the induction of cytokine and the activation of dendritic cell (DC). In addition, immunization of mice with sal-FliC causes a robust activation of immune cells (29). In some published studies, purified *Salmonella* flagellins were intraperitoneally injected into mice, then the spleen, ileal tissues, and serum were collected after 2, 4, 6, 8, and 24 h. The expression of Th17-related cytokines at different time points showed that the immune response induced by *Salmonella* flagellin was temporary and peaked at 2 h, and cytokine expression levels returned to baseline after 24 h (40, 41). Interestingly, in this study, we found that similar to the immunization of mice with sal-FliC, mice immunized with SFB flagellins promote the swift, intense, transient production of the factors controlling Th17 differentiation and Th17-related cytokines, such as IL-17A, IL-17F, and IL-22. And SFB flagellin administration significantly triggered the upregulation of activator protein 1 transcription factor mRNA level, which contributes to the differentiation of Th17 cells (42). However, there were no significant changes in the expression of genes encoding IL-23, IL-21, and TGF-β in our *in vitro* cell co-culture experiments, and the expression of IL-21 and TGF-β slightly changed in our *in vivo* experiments. Cytokines which promoted the differentiation of Th17 cells have been well characterized *in vitro*. IL-6, TGF-β, and IL-21 are known to promote the differentiation of Th17 cells. The coordinated activities of IL-1 and TNF can accelerate this process. However, studies have proved that according to the location and timing of the immune response, the collaboration of pro-inflammatory cytokines such as IL-21, TNF, and IL-1 with TGF-β, might play contradictory pro- or anti-inflammatory roles (43, 44), which also explains the inconsistent expression of some cytokines *in vivo* and *in vitro*. Interestingly, among the Th17-conducting cytokines, IL-1β was upregulated, whether in cocultures of CD4+ T cells and CD11c+ cells stimulated with SFB flagellins or in the gut of mice treated with SFB flagellins, which is concordant with the findings of previous reports (16, 35). IL-1β induced by commensal microbiota is vital for the development of TH17 cells (16). Atarashi et al. (35) showed that the secretion of SAAs induced by SFB adhesion acted on CD11c+ cells to stimulate the significant production of IL-1β and other cytokines, which then caused constitutive accumulation of Th17 cells. Inadequate IL-1β production is part of the reason for the reduction in Th17 cell accumulation.

A recent study has shown contradictory results on the effect of flagellin (45), suggesting that flagellin can increase the production of Treg cells and may inhibit a Th17-like immune response. However, this study was based an experiment in which flagellin doses were much higher than those used in the present study, and instead of SFB, their flagellin originated from *Vibrio vulnificus*, in which the role of flagellins remains unclear. In addition, low and high doses of LPS, the TLR4 ligand, promote Th2 and Th1 responses, separately (46). Thus, we also speculate that the different influences of flagellin might be depend on different doses administered to the mice. Although the role of bacterial flagellins in shaping the host immune response has also been reported (47), the protein sequence similarity between SFB and other bacterial flagellins is very low and information on the contribution of SFB flagellins to the immune system is limited due to its difficulty in electron microscope analysis. Our research group was the first to demonstrate that SFB flagellins are expressed in the ileum mucosa (31). In this study, we proved that SFB flagellins could promote the differentiation of Th17 cells and assessed its regulatory network. Consistent with SFB, SFB flagellins have an intimate relationship with IECs, reflected in the SI EC gene expression profiles induced by SFB flagellins administration. The expression of several SI EC specific genes and inflammatory response genes were highly upregulated, including Duox2, Duoxa2, SAA3, Tat, and Lcn2. Studies have shown SFB promoted the production of reactive oxygen species (ROS), which can suppress the activity of Rho GTPase family and then affect actin reorganization through adhesion to IECs (35, 48). In this study, SFB flagellins administration caused a marked increase expression of Duox2 and Duoxa2. Thus, we believe that SFB flagellins may be involved in the reorganization of actin. In addition, SFB induce SI EC to produce SAAs and then evoke the induction of IL-17. Interestingly, SFB flagellins elicited increase mRNA levels of SAA2 and SAA3, but not SAA1. However, we found that the mRNA levels of C/EBPD was up-regulated significantly. Consistent with this result, the co-induction of SAA3 and C/EBPD expression was observed in MODE-K cells stimulated by SFB flagellins *in vitro*. However, host specificity of SFB flagellins in immune stimulation was not observed either *in vivo* or *in vitro*.

Although SFB flagellins were not detected by electron microscope analysis, the expression of SFB flagellins in the intestine has been proven by a variety of technologies. In addition, the genome sequences showed that SFB encode more than 40 (3% of total) putative chemotaxis- and flagella-related proteins (19, 30). In addition, comparative genomic analysis showed that SFB genomes were similar to *Clostridial* genomes and several *Clostridial* genomes also encoded flagellar assembly proteins (20). Surprisingly, only SFB flagellin proteins have the TLR5 binding sites (30). Our previous results constitute evidence, which is consistent with another study (19), that SFB encode four types of flagellin, three of which, FilC2, FliC3, FliC4, were recognized by Toll-like receptor 5. In this study, western blot analysis showed that CD4+ T cells, splenic CD11c+ cells, and a mouse SI EC line (MODE-K) all expressed TLR5, with the SI EC line showing the highest expression (Supplementary Figure 7A). Next, we investigated whether TLR5 in CD4+ T cells and splenic CD11c+ cells, as classic costimulatory receptor, play an important role in promoting T cell activation. We stimulated T cells alone with SFB flagellins to determine whether there is an interplay between TLR5 and TCR during gut T cell activation. We found that the addition of SFB flagellins to cultures with only T cells, did not promote the differentiation of Th17 cells. Then, the anti-TLR5 blocking antibody was introduced into the cocultures of CD4+ T and CD11c+ cells. Surprisingly, the effect of T cell activation was not significantly attenuated when the anti-TLR5 blocking antibody was added (Supplementary Figure 7B). These findings suggest that SFB flagellins promote T cell activation and this effect may not or at least not only correlate with TLR5 activation. Most importantly, studies have shown only a slight reduction in the percentage of Th17 cells in the lamina propria of both MyD88- and TRIF-deficient mice, demonstrating that TLR signaling may be dispensable for Th17 cell differentiation in the SI LP (39). So we speculate that the TLR5 signaling pathway may be one, but not the only one, of the unique signaling pathways elicited by SFB colonization. Recent work has highlighted that SFB tightly adheres to SI ECs and transferred SFB antigenic proteins into SI ECs by microbial adhesion-triggered endocytosis (MATE), thus participating in host immune response (17). However, it is still unclear which components of SFB may be involved in this process. 3D reconstruction of SFB hook-like holdfast revealed membrane vesicles originate at the tips of holdfast (17). One speculated that the tail-like structures might be, in fact, flagella (49). However, the involvement of SFB flagellins in endocytosis of SFB by epithelial cells and its occurrence in MATE vesicles remain elusive.

SFB have been extensively studied due to their unique ability to drive the generation of intestinal Th17 cells. However, functionally analogous microbes have not been identified to date. A recent report showed that a human symbiont bacterial species called *Bifidobacterium adolescentis* could induce the accumulation of intestinal Th17 cells in mice (50). Interestingly, another study also showed that a coalition of 20 symbionts from an IBD patient could promote the production of Th17 cells in mice (35). This suggests alternative pathways of promoting Th17 cell accumulation. In this study, we demonstrated for the first time that SFB flagellins as SFB antigens could promote the differentiation of Th17 cells. However, another investigation has shown that SFB protein P3340 as antigen could also promote Th17 cell differentiation (6). This thus suggests that, rather than a single bacterial component, multiple bacterial components such as SFB flagellins and SFB protein P3340 coordinate to facilitate Th17 cell differentiation.

In summary, while flagellins are well-acknowledged as a potent activator of a broad range of cell types involved in innate and adaptive immunity, little is known about the contribution of SFB flagellins in immunity. In this study, we found that SFB flagellins have the similar immune function as SFB, influencing the expression of the same genes, including Th17-related cytokines, such as IL-6, IL-1β, TNF-α, IL-17A, IL-17F, and IL-22, and epithelial cell-specific genes, such as Duox2, Duoxa2, SAA3, Tat, and Lcn2. Thus, by linking SFB flagellins to the expression of these genes, we present a new viewpoint in which SFB flagellins, as a previously unappreciated component, are a key of the immune response to SFB. However, a critical question remains: How do SFB flagellins promote the differentiation of Th17 cells? Further investigations of such questions may have a tremendous impact on our understanding of the molecular and cellular mechanisms that SFB regulates in intestinal T cell homeostasis.

## Data Availability Statement

The datasets generated for this study can be found in NCBI Sequence Read Archive, https://www.ncbi.nlm.nih.gov/bioproject/PRJNA531884/.

## Ethics Statement

This study was carried out in accordance with the recommendations of Guide for the Care and Use of Laboratory Animals published by the National Institutes of Health. The protocol was approved by the Laboratory Animal Care and Usage Committee of Zhejiang University.

## Author Contributions

YWa and YY designed the experiments, performed most of the experiments, and analyzed the data. YWa analyzed the data and wrote the manuscript. YZ, XC, and YWu helped in some of the mouse experiments. XW and YL helped with the statistical analyses of some data. CX and HC conceived and supervised this study, provided critical suggestions and discussions throughout the study, and revised the manuscript. All authors have read and approved the final manuscript.

### Conflict of Interest

The authors declare that the research was conducted in the absence of any commercial or financial relationships that could be construed as a potential conflict of interest.
