# Supplementary material for: Induction of Intestinal Th17 Cells by Flagellins From Segmented Filamentous Bacteria
Source: Front Immunol. 2019 Nov 22;10:2750. doi: 10.3389/fimmu.2019.02750 (PMC6883716; doi:10.3389/fimmu.2019.02750)

**Supplementary materials for**

**Induction of intestinal Th17 cells by flagellins from** **segmented filamentous bacteria**

**Yanling Wang^1^, Yeshi Yin^2^, Xin Chen^1^, Yongjia Zhao^1^, Yichen Wu^1^, Yifei Li^1^, Xin Wang^3^, Huahai Chen^2, *^, Charlie Xiang^1, *^**

^1^State Key Laboratory for Diagnosis and Treatment of Infectious Diseases, National Clinical Research Center for Infectious Diseases, Collaborative Innovation Center for Diagnosis and Treatment of Infectious Diseases, The First Affiliated Hospital, College of Medicine, Zhejiang University, Hangzhou, Zhejiang, People's Republic of China;

^2^Key Laboratory of Comprehensive Utilization of Advantage Plants Resources in Hunan South, College of Chemistry and Bioengineering, Hunan University of Science and Engineering, Yongzhou, Hunan, People's Republic of China;

^3^State Key Laboratory of Breeding Base for Zhejiang Sustainable Pest and Key Laboratory for Food Microbial Technology of Zhejiang Province, Zhejiang Academy of Agricultural Sciences, Hangzhou, Zhejiang, People's Republic of China

***Correspondence:**

Huahai Chen

[chenhuahai2008@163.com](mailto:chenhuahai2008@163.com)

Charlie Xiang

[cxiang@zju.edu.cn](mailto:cxiang@zju.edu.cn)

**Keywords: Segmented filamentous bacteria, flagellin, Th17 Cells, IL-17A,** **SI EC**

# Supplementary Table 1. Primers Used for Real-Time PCR

| **Primer Name** | **Sequence (5’–3’)** | **Species** |
| --- | --- | --- |
| Gapdh-Forward  Gapdh-Reverse | GTCGTGGAGTCTACTGGTGTCTTC  GTCATATTTCTCGTGGTTCACACC | Mouse |
| β-actin-Forward  β-actin-Reverse | CCTGAGCGCAAGTACTCTGTGT  GCTGATCCACATCTGCTGGAA | Mouse |
| IL-17A-Forward  IL-17A-Reverse | GGACTCTCCACCGCAATGA  GGCACTGAGCTTCCCAGATC | Mouse |
| IL-17F-Forward  IL-17F-Reverse | TGCTACTGTTGATGTTGGGAC  AATGCCCTGGTTTTGGTTGAA | Mouse |
| IL-6-Forward  IL-6-Reverse | ACAACCACGGCCTTCCCTACTT  CACGATTTCCCAGAGAACATGTG | Mouse |
| IL-1β-Forward  IL-1β-Reverse | TGAAGTTGACGGACCCCAAA  TGATGTGCTGCTGTGAGATT | Mouse |
| IL-21-Forward  IL-21-Reverse | GGCAATGAAAGCCTGTGGAA  GGCAATGAAAGCCTGTGGAA | Mouse |
| IL-23-Forward  IL-23-Reverse | CACCTCCCTACTAGGACTCAGC TGGGCATCTGTTGGGTCT | Mouse |
| IL-22-Forward  IL-22-Reverse | ATGAGTTTTTCCCTTATGGGGAC  GCTGGAAGTTGGACACCTCAA | Mouse |
| TNF-α-Forward  TNF-α-Reverse | CCCCAAAGGGATGAGAAGTTC  GCTTGTCACTCGAATTTTGAGAA | Mouse |
| TGF-β-Forward  TGF-β-Reverse | CTCCCGTGGCTTCTAGTGC  GCCTTAGTTTGGACAGGATCTG | Mouse |
| AhR-Forward  AhR-Reverse | AGCCGGTGCAGAAAACAGTAA  AGGCGGTCTAACTCTGTGTTC | Mouse |
| RORγt-Forward  RORγt-Reverse | CCGCTGAGAGGGCTTCAC  TGCAGGAGTAGGCCACATTACA | Mouse |
| Saa1-Forward  Saa1-Reverse | CATTTGTTCACGAGGCTTTCC  GTTTTTCCAGTTAGCTTCCTTCATGT | Mouse |
| Saa2-Forward  Saa2-Reverse | TGCAAGAGAGAGCTTTCAGG  TCAGTATTTGGCAGGCAGTC | Mouse |
| Saa3-Forward  Saa3-Reverse | GCAACTACTGGGTTGAGATA  ATTCAGCACATTGGGATG | Mouse |
| Duoxa2-Forward  Duoxa2-Reverse | ACCGCTGCTCATTGTTATCC  AGTGCACAGCCACAATTTCG | Mouse |
| Duox2-Forward  Duox2-Reverse | TGCGCCTGTTACTGTGATTG  AATGGAAAGCAGCAGACAGC | Mouse |
| Cebpd-Forward  Cebpd-Reverse | GGCACAGTCCGAGAAAAGG  CTTCTGCTGCATCTCCTGGT | Mouse |
| IFN-γ-Forward  IFN-γ- Reverse | CTTCTTCAGCAACAGCAAGG  TGAGCTCATTGAATGCTTGG | Mouse |
| T-bet- Forward  T-bet-Reverse | CAACAACCCCTTTGCCAAAG  TCCCCCAAGCAGTTGACAGT | Mouse |

**Supplementary Figure 1. The expression of Th17**-**related cytokines in cocultures of CD4+ T cells and CD11c+ cells stimulated with SFB flagellins.** (A), Activation of SILP CD4+ T cells from WT C57BL/6 mice by SFB-mFliC3, contaminating *Escherichia coli* proteins from chemically competent BL21(DE3) cells or PBS. IL-17A ELISA assay was evaluated after 72 h. a-d, represent statistical significance relative to the control group; SFB-mFliC3 group; BL21 proteins (1 μg/mL) group; BL21 proteins (5 μg/mL) group respectively. (**B**), SILP CD4+ T cells from WT C57BL/6 mice were cocultured with CD11c+ cells and treated with SFB-mFliC3, SFB-rFliC3, SFB-m5i-FliC3, sal-FliC3, or PBS for 0 h, 24 h, 48 h, 72 h, and 96 h. Cell viability was determined using the CCK-8 assay. (C), The mRNA expression of IL-23, IL-21, and TGF-β relative to Gapdh in a co-culture system of the SILP CD4+ T cells and CD11c+ cells stimulated with SFB flagellins. (D), The mRNA expression of IFN-γ and T-bet relative to Gapdh in a co-culture system. (E), Activation of SILP CD4+ T cells from mice by SFB flagellins, sal-FliC3 or PBS. IFN-γ ELISA assay was evaluated after 72 h. Data are expressed as mean ± SEM from three independent experiments. Error bars indicate median values. a-e, represent statistical significance relative to the control group; mFliC3 group; m5i-FliC3 group; rFliC3 group; sal-FliC3 group respectively.

**
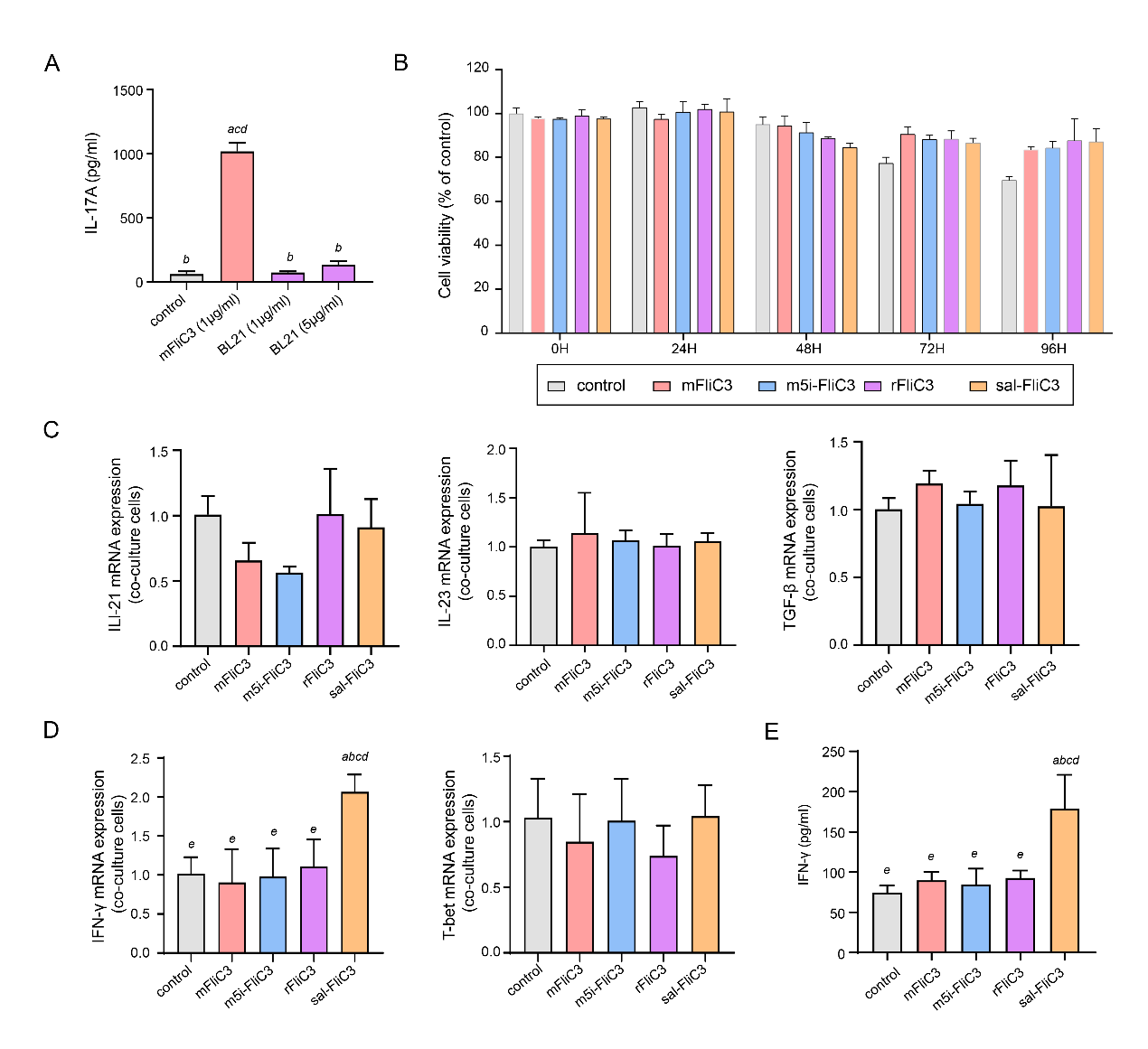
**

**Supplementary Figure 2.** **Th17-related cytokine return to normal levels** **in mice treated with flagellins after stimulation for 24 h.** C57BL/6 mice (n = 6) were treated i.p. with SFB flagellins or sal-FliC3. The distal ileum and serum cytokine were assayed 24 h later for the quantification of mRNA levels and ELISA. (**A**), Serum and intestinal IL-17A concentrations in conventional and experimental mice that received i.p. immunizations for 24 h (n=6). (**B**), Serum and intestinal IL-6 concentrations in conventional and experimental mice that received i.p. immunizations for 24 h (n=6). Error bars in panels A and B indicate median values. a-f, represent statistical significance relative to the control group; anti-CD3 group; mFliC3 group; m5i-FliC3 group; rFliC3 group; sal-FliC3 group respectively. (**C**), the mRNA expression of IL-17A and IL-6 relative to Gapdh in intestines of the control group and experimental mice that received i.p. immunizations for 24 h.


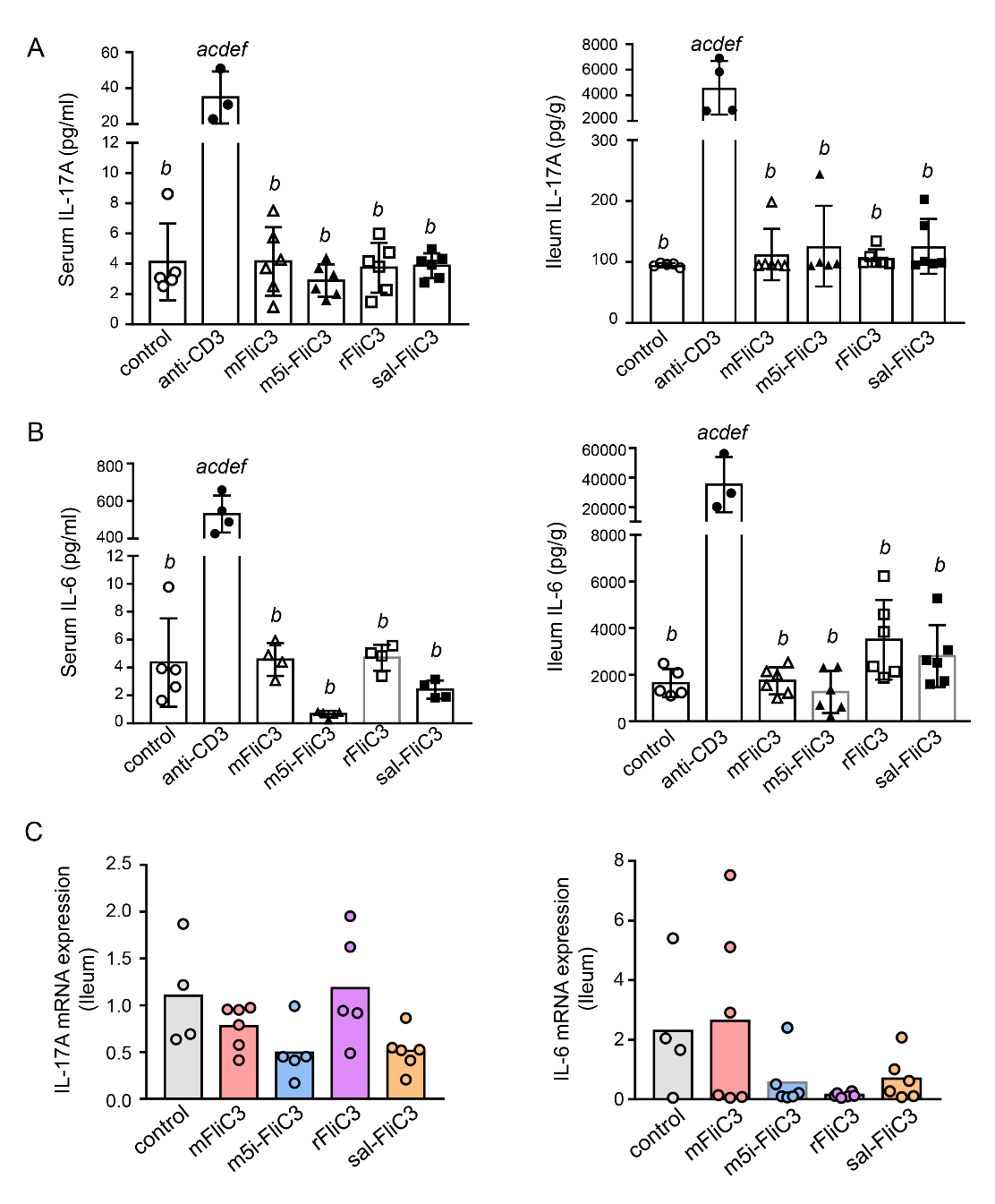


**Supplementary Figure 3. Immune system-related transcriptional programs induced by SFB flagellins.** Heatmap generated from immune system-related genes significantly expressed (p < 0.05) between all of the treatments after 2 h. Each line represents a specific gene of interest and each column represents a single mouse. Green, genes that were at least two-fold downregulated; red, genes that were at least two-fold upregulated.


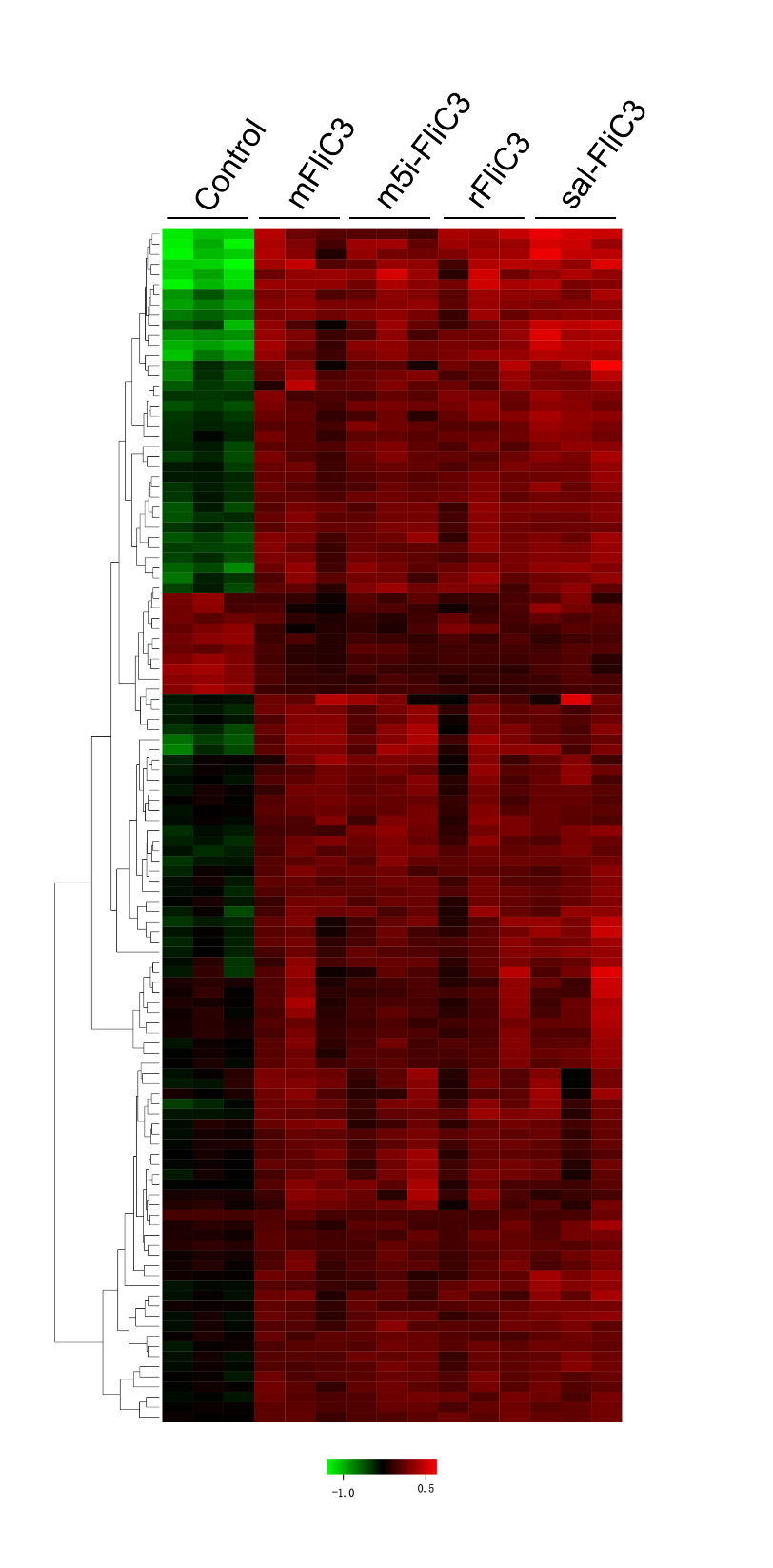


**Supplementary Figure 4. Identification of upregulated genes in IL-17 signaling pathway related to SFB flagellins.** The comparisons of gene expression specific for IL-17 signaling pathway arranged by fold change in the control and experimental groups after RNA-seq.


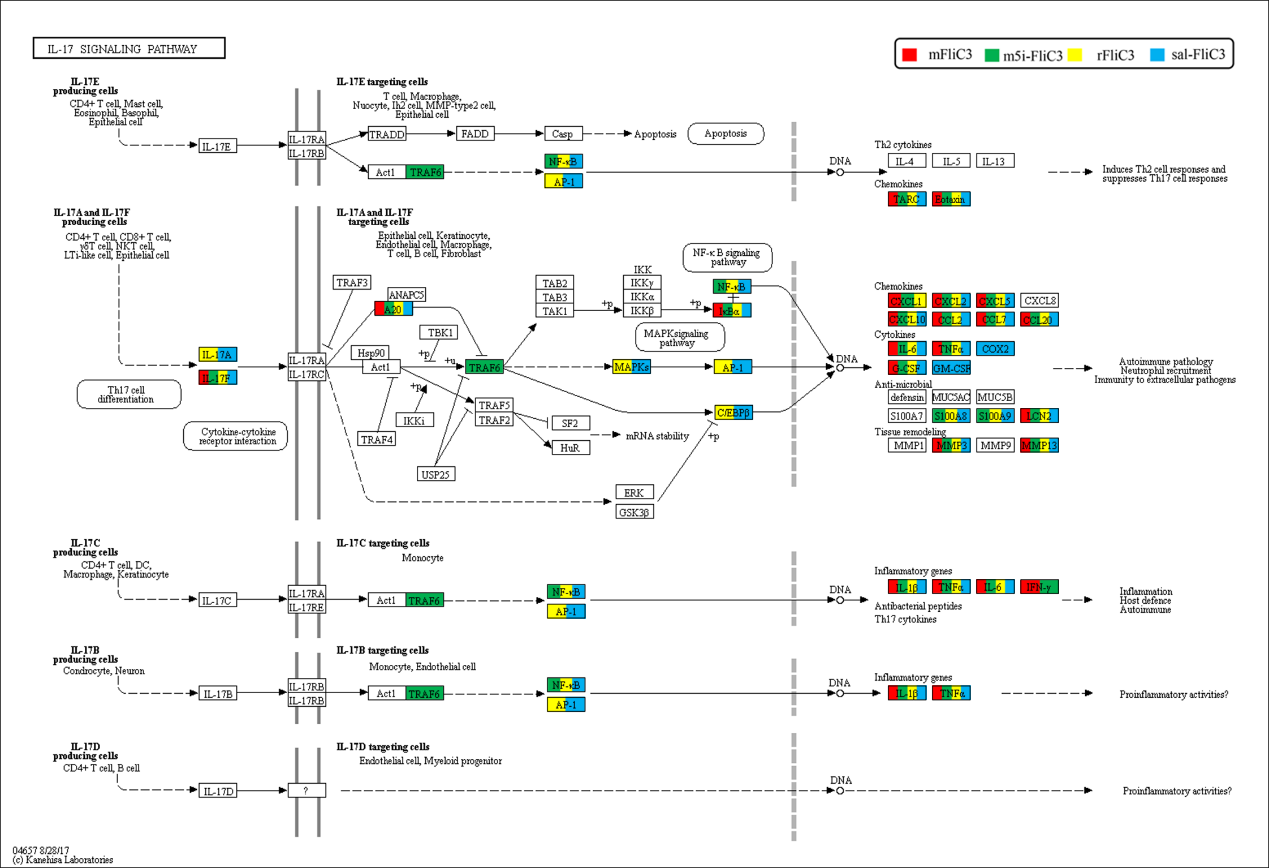


**Supplementary Figure 5. Quantitative analysis of cytokines related to Th17 cells in the mice with SFB flagellins administration.** (**A**), qPCR for IL-23 and AhR relative to Gapdh in the intestines of the indicated mice, which were i.p. injected with SFB flagellins or other antigens for 2 h (n=6). (**B**), qPCR for IL-17F, IL-22, IL-1β, and IFN-γ relative to Gapdh in intestinal from the indicated mice, which were i.p. injected with SFB flagellins or other antigens for 24 h (n=6). (C), qPCR for IL-17A, IL-17F, IL-21, IL-22, IL-6, and TNF-α relative to Gapdh in the spleen from the indicated mice that were i.p. injected with SFB flagellins or other antigens for 2 h (n=3). Error bars indicate the median values.

**
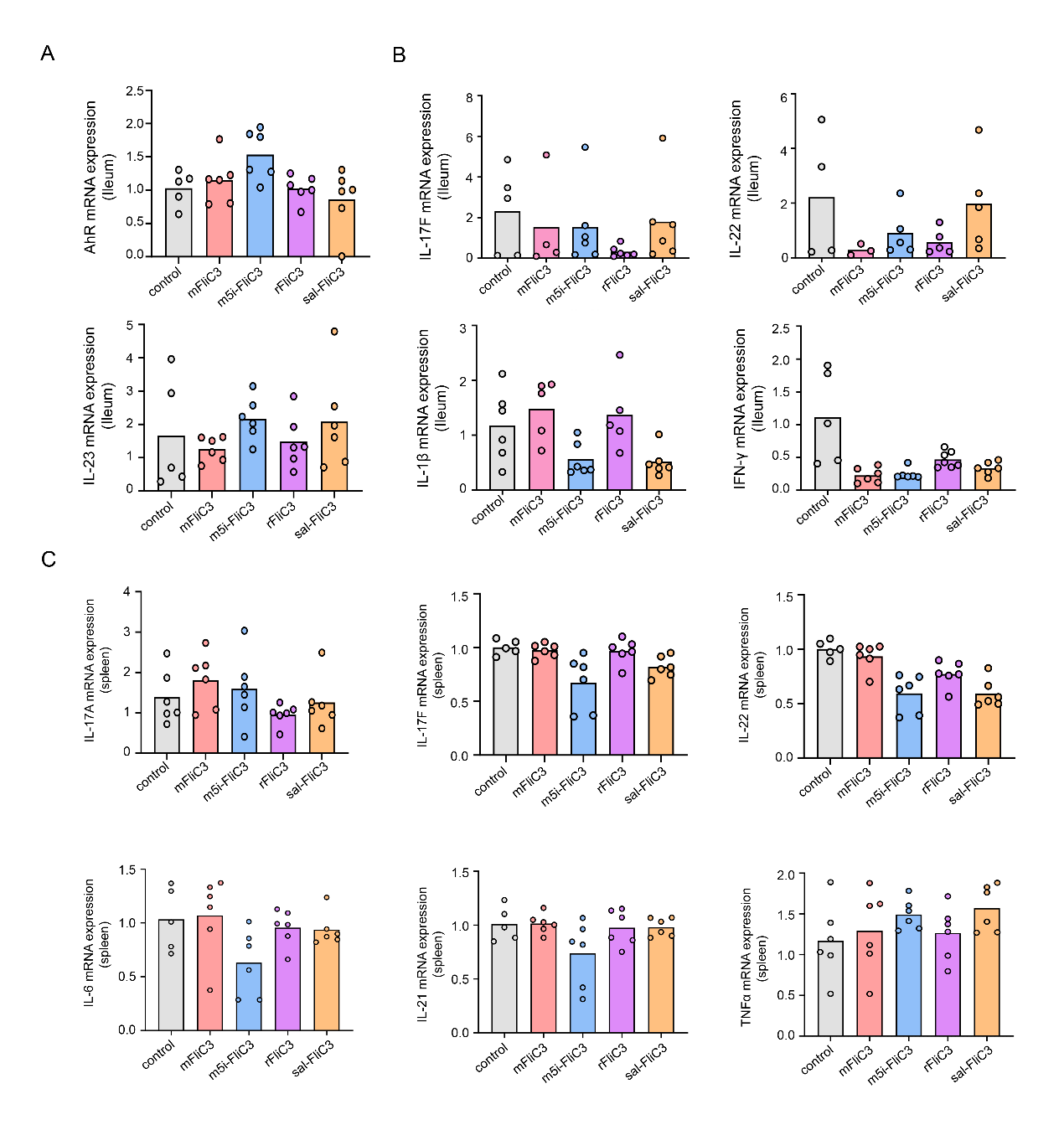
**

**Supplementary Figure 6. Quantitative analysis of SAA1 and SAA2 in IEC stimulated by SFB flagellins.** MODE-K cells were stimulated by SFB flagellins for 24 h. The cells were harvested, and SAA1 and SAA2 mRNA expression levels were evaluated by qPCR. Data are expressed as the mean ± SEM of three independent experiments.


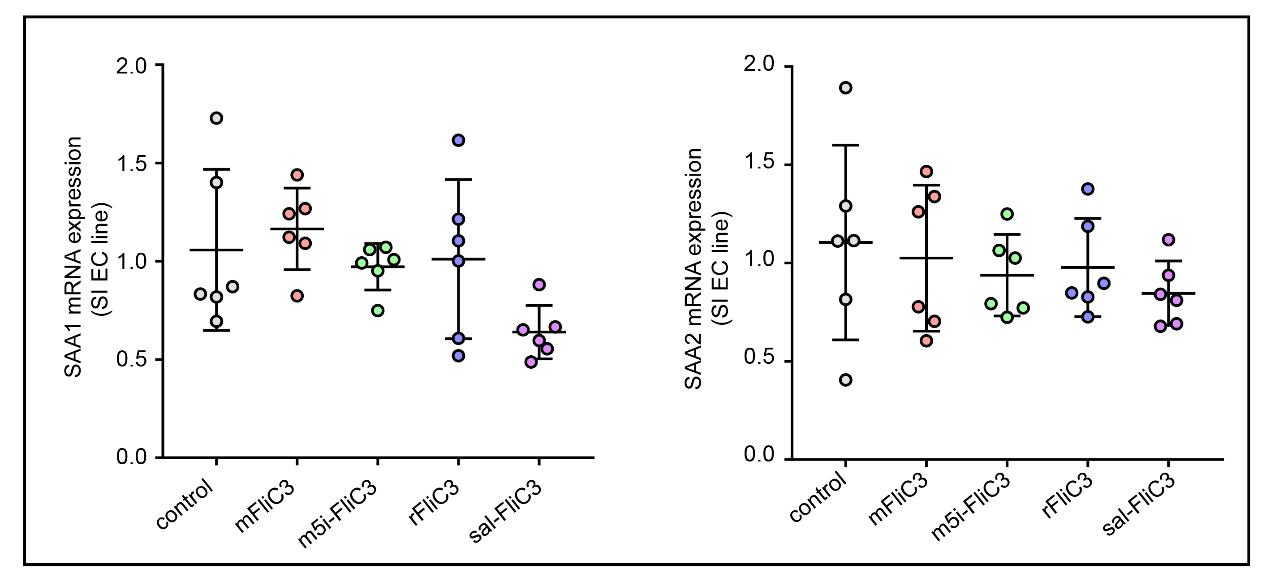


**Supplementary Figure 7. T cell activation promoted by SFB flagellins is independent of TLR5.** (**A**), Western blot assay indicates that protein expression of TLR5 in SILP CD4+ T cell, splenic CD11c+ cell, and mouse SI EC line (MODE-K). (**B**), Purified CD4 T cells and CD11c+ cells were pre-incubated with an anti-TLR5 monoclonal antibody or the same concentration of monoclonal Rat IgG (InvivoGen, USA) for 1 h. The cells were then treated with SFB flagellin proteins at 37°C for three days. The conditioned media were collected and analyzed for IL-17A using an ELISA kit.


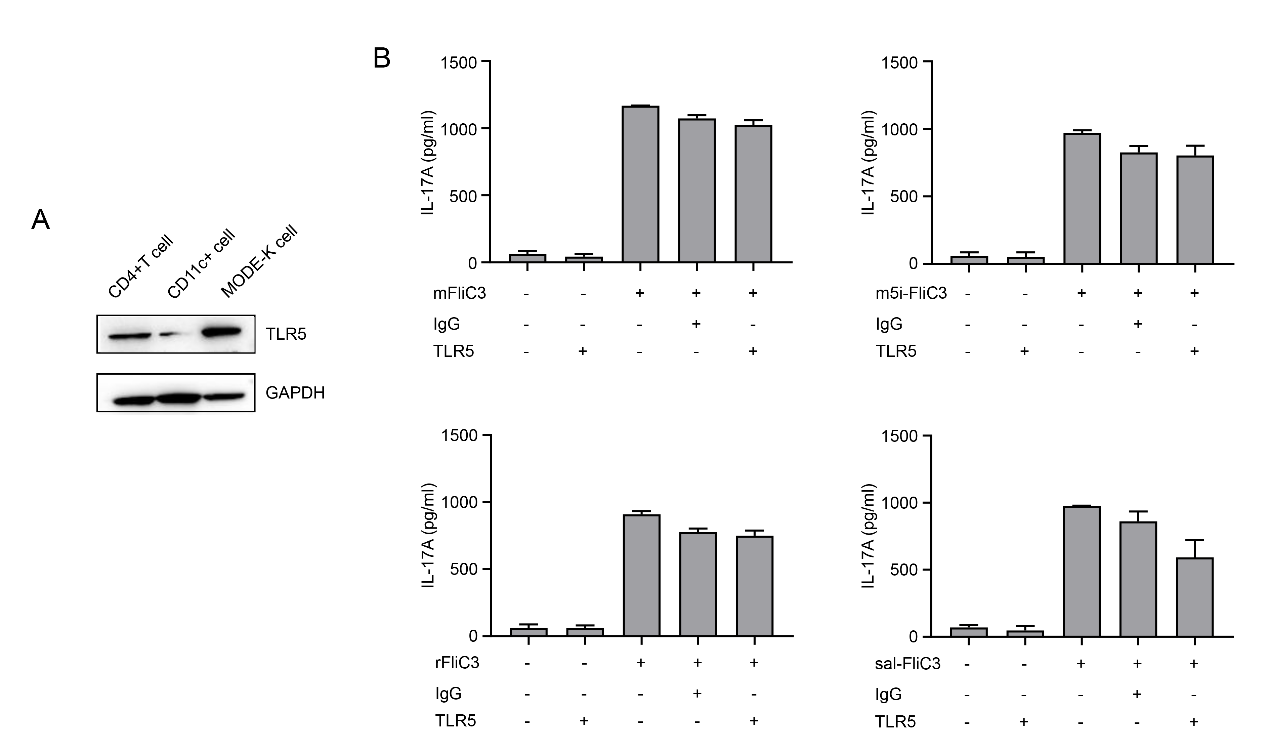

Supplement: Supplementary file 1 [file Data_Sheet_1.docx]
